# Molecular relapse after first-line intensive therapy in patients with CBF or *NPM1*-mutated acute myeloid leukemia – a FILO study

**DOI:** 10.1038/s41375-024-02335-2

**Published:** 2024-07-17

**Authors:** Corentin Orvain, Sarah Bertoli, Pierre Peterlin, Yohann Desbrosses, Pierre-Yves Dumas, Alexandre Iat, Marie-Anne Hospital, Martin Carre, Emmanuelle Tavernier, Jérémie Riou, Anne Bouvier, Audrey Bidet, Sylvie Tondeur, Florian Renosi, Marie-Joelle Mozziconacci, Pascale Flandrin-Gresta, Bérengère Dadone-Montaudié, Eric Delabesse, Arnaud Pigneux, Mathilde Hunault-Berger, Christian Recher

**Affiliations:** 1 Maladies du Sang, CHU d’Angers, Angers, France; 2 Fédération Hospitalo-Universitaire Grand-Ouest Acute Leukemia, FHU-GOAL, Nantes, France; 3 Université d’Angers, Inserm UMR 1307, CNRS UMR 6075, Nantes Université, CRCI2NA, F-49000 Angers, France; 4 Hématologie Clinique, CHU de Toulouse, Institut Universitaire du Cancer de Toulouse Oncopole, Toulouse, France; 5 Hématologie Clinique, CHU de Nantes, Nantes, France; 6 Hematologie Clinique, CHU de Besançon, Besançon, France; 7 Hématologie Clinique, CHU de Bordeaux, Bordeaux, France; 8 Hématologie Clinique, CHU de Nice, Nice, France; 9 Hématologie Clinique, Institut Paoli-Calmettes, Marseille, France; 10 Hématologie Clinique, CHU de Grenoble, Grenoble, France; 11 Hématologie Clinique, Institut de Cancérologie Lucien Neuwirth, Saint-Priest-en-Jarez, France; 12 Univ Angers, CHU Angers, Inserm, CNRS, MINT, SFR ICAT Angers, France; 13 Laboratoire d’Hématologie, CHU d’Angers, Angers, France; 14 Laboratoire d’Hématologie, CHU de Bordeaux, Bordeaux, France; 15 Grenoble Alpes University, University Hospital, Hematology Molecular Biology department, Grenoble, France; 16 Laboratoire d’Hématologie et d’Immunologie Cellulaire, CHU de Besançon, Besançon, France; 17 Biopathologie, Institut Paoli-Calmettes, Marseille, France; 18 Laboratoire d’Hématologie, CHU de Saint Etienne, Saint Etienne, France; 19 Laboratoire d’Oncologie Moléculaire, CHU de Nice, Nice, France; 20 Laboratoire d’Hématologie, CHU de Toulouse, Institut Universitaire du Cancer de Toulouse Oncopole, Toulouse, France

**Keywords:** Epidemiology, Acute myeloid leukaemia

## Abstract

Patients with Core-Binding Factor (CBF) and *NPM1*-mutated acute myeloid leukemia (AML) can be monitored by quantitative PCR after having achieved first complete remission (CR) to detect morphologic relapse and drive preemptive therapy. How to best manage these patients is unknown. We retrospectively analyzed 303 patients with CBF and *NPM1*-mutated AML, aged 18–60 years, without allogeneic hematopoietic cell transplantation (HCT) in first CR, with molecular monitoring after first-line intensive therapy. Among these patients, 153 (51%) never relapsed, 95 (31%) had molecular relapse (53 received preemptive therapy and 42 progressed to morphologic relapse at salvage therapy), and 55 (18%) had upfront morphologic relapse. Patients who received preemptive therapy had higher OS than those who received salvage therapy after having progressed from molecular to morphologic relapse and those with upfront morphologic relapse (three-year OS: 78% *vs*. 51% *vs*. 51%, respectively, *P* = 0.01). Preemptive therapy included upfront allogeneic HCT (*n* = 19), intensive chemotherapy (*n* = 21), and non-intensive therapy (*n* = 13; three-year OS: 92% *vs*. 79% *vs*. 58%, respectively, *P* = 0.09). Although not definitive due to the non-randomized allocation of patients to different treatment strategies at relapse, our study suggests that molecular monitoring should be considered during follow-up to start preemptive therapy before overt morphologic relapse.

## Introduction

Patients with Core-Binding Factor (CBF), characterized by the t(8;21) translocation and inversion inv(16)/t(16;16), and their molecular equivalents, *RUNX1*::*RUNX1T1* and *CBFB*::*MYH11*, and *NPM1*-mutated acute myeloid leukemia (AML) are considered as favorable or intermediate-risk according to the 2022 European LeukemiaNet (ELN) classification [1]. Allogeneic hematopoietic cell transplantation (HCT) is usually not considered in those achieving first complete remission (CR) with good molecular response [2, 3]. In the 20–30% of patients with CBF or *NPM1*-mutated AML who relapse, chemosensitivity is usually maintained and a second CR can be obtained in 50 to 80% of patients after salvage therapy, with consolidative allogeneic HCT being associated with better overall survival (OS) [4–8].

Many studies have shown the utility of molecular follow-up of CBF transcripts and *NPM1* mutations after first CR to detect emerging relapse [2, 9–14] and close molecular monitoring by reverse transcriptase quantitative PCR (RT-qPCR) or digital PCR is now recommended [15]. Because molecular relapse can precede overt morphologic relapse by a few weeks to a few months, preemptive therapy, given at the time of molecular relapse, can be considered to prevent morphologic relapse [16–18]. It is however unclear if molecular monitoring can give sufficient lead-in time to intervene [19] and if therapy given at the time of MRD relapse can improve tolerance and efficacy of salvage therapy [14, 17]. In acute promyelocytic leukemia, retrospective studies have shown that early therapeutic intervention in patients with molecular relapse is associated with a better outcome in comparison to delaying treatment until morphologic relapse [20, 21]. Due to the lack of data from large series or clinical trials in non-APL patients, there is no recommendation on how to best manage these patients.

To explore if patients have better outcomes when treated at the time of molecular relapse, rather than after overt morphologic relapse, we retrospectively analyzed the outcome of 303 CBF or *NPM1*-mutated AML patients treated by intensive chemotherapy who had MRD monitoring after having achieved first CR. Treatment modalities (and outcomes) after molecular and morphologic relapse were also explored.

## Methods

### Study cohort

All CBF and *NPM1*-mutated AML patients, aged 18–60 years, diagnosed between 2010 and 2019, who had MRD molecular monitoring (RT-qPCR of *RUNX1::RUNXT1* or *CBFB::MYH1* transcripts or classical *NPM1* mutations [A, B, and D]) on either blood or bone marrow samples, as available, after first-line therapy including induction therapy with daunorubicin (60–90 mg/m² on days 1–3) or idarubicin (8–9 mg/m² on days 1–5) and cytarabine (200 mg/m² on days 1–7) followed by intermediate (1.5 g/m²/12 h, three days) or high-dose (3 g/m²/12 h, three days) cytarabine consolidation, without allogeneic HCT in first CR, in our nine centers were included. Based on previous studies [2, 3], patients not reaching at least a 3-log reduction in bone marrow (for CBF transcripts ; starting in 2006) or a 4-log reduction in blood (for *NPM1* mutations ; starting in 2013) in MRD before initiation of the second consolidation cycle were candidates for allogeneic SCT in first CR if they had a matched related or unrelated donor. Patients with measurable CBF transcripts or *NPM1* mutations at the end of consolidation therapy were included given they remained in morphologic CR at the end of treatment. Although molecular follow-up is standard-of-care for patients with CBF AML since the CBF-2006 trial [2], completed in 2010, and *NPM1*-mutated patients since the start of the BIG-1 study (NCT02416388), ongoing since 2015, we cannot exclude that some patients did not have molecular follow-up. The study was registered on ClinTrialGov (NCT04931992). Follow-up was current as of February 10, 2023.

### Data collection

Data were collected from individual medical files including baseline characteristics, MRD follow-up, treatment used at relapse, and outcomes. The 2022 European LeukemiaNet (ELN) criteria were used to assign cytogenetic risk at diagnosis [1]. Secondary AML was defined as disease following an antecedent hematologic disorder (AHD; i.e., MDS, myeloproliferative neoplasm [NPM], and MDS/MPN such as chronic myelomonocytic leukemia [CMML]) or treatment with systemic chemotherapy and/or radiotherapy for a different disorder [22, 23]. MRD responses during first-line treatment were reported as log-reduction after induction therapy, best log reduction during treatment, and as the ELN MRD response categories (CR_MRD-_, CR_MRD-LL_ at low level [qPCR <2%], or CR_MRD+_ other than CR_MRD-LL_) [15], in both bone marrow (BM) and/or peripheral blood (PB), as available.

After completion of consolidation therapy, MRD measurements were generally performed every three months by quantitative RT-PCR during follow-up for 24 months in either bone marrow (BM) or peripheral blood (PB) in eight different reference laboratories according to international guidelines [15]. Although there was no standardization for MRD assessment, all participating laboratories regularly carry out the same quality controls within the cooperative group, GBHM (‘Groupes des Biologistes Moléculaires des Hémopathies malignes’). The detection limit for the assays used was 10^−^^4^ throughout the study period. Molecular relapse was defined according to ELN recommendations as a conversion from MRD negativity to positivity or an increase in MRD normalized copy numbers >1 log between two positive samples [15]. All molecular relapses were confirmed on a second sample (day of first sample was used to define molecular relapse). Morphologic relapse was defined as bone marrow blasts ≥ 5%, reappearance of blasts in the blood in at least two peripheral blood samples at least one week apart, or development of extramedullary disease [1]. Bone marrow evaluation was done in all patients receiving preemptive therapy to rule out morphologic relapse.

Treatment after relapse was given at the discretion of the attending physician and was recorded and classified as intensive (high-dose cytarabine-containing regimens, “3 + 7” or equivalent), non-intensive (azacitidine, small molecule inhibitor), or upfront allogeneic HCT (e.g., without prior salvage therapy). Some patients received transplantation with conditioning regimens using intensive chemotherapy followed by reduced intensity conditioning [24]. Status (molecular or morphologic relapse) at first salvage therapy initiation was also recorded, as was the best response after each salvage treatment course.

### Statistical analysis

Categorical variables were presented as numbers with proportions and compared using the Chi² test or the Fisher’s exact test, for small samples (expected values < 5). Continuous variables were presented as medians with interquartile range (IQR) and compared using the non-parametric Mann and Whitney test. The Pearson correlation test was used to calculate correlation between transcript ratio in BM and PB at different time points (i.e., after induction therapy, best response, and at molecular relapse). Unadjusted probabilities of overall survival (OS; event: death) were estimated using the Kaplan-Meier method and compared with the Log-Rank test; associations with OS were assessed using Cox regression. Probabilities of molecular and morphologic relapse and treatment-related mortality (i.e., mortality not due to disease) were summarized using cumulative incidence estimates. A multistate model was used to calculate the predicted probabilities of being in a specific state during follow-up. All tests were two-sided with a significant level *P* < 0.05. Statistical analyses were performed with R (R Foundation for Statistical Computing, Vienna, Austria; http://www.r-project.org).

## Results

### Patient characteristics

**Fig. 1:**
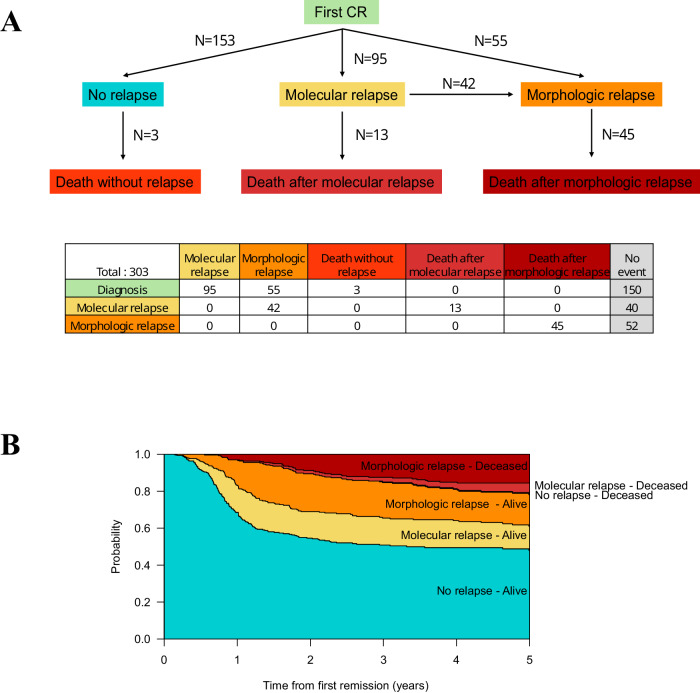
Description of states and transitions in the multistate model. **A** Flow chart of patients with CBF and *NPM1*-mutated AML included in the study cohort with description of states and transitions used in the multistate model. **B** Predicted probabilities of being in a specific state over time, from initial diagnosis.

**Fig. 2:**
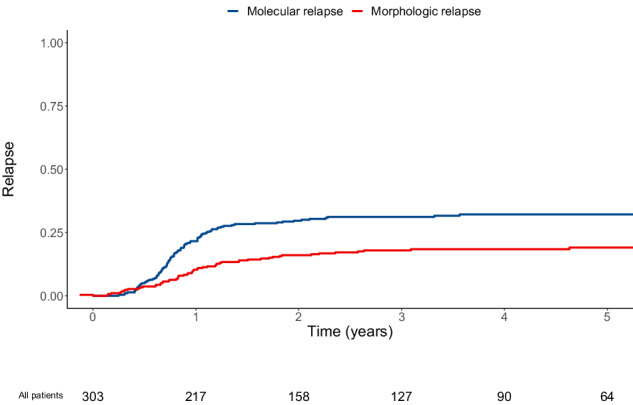
Cumulative incidence of molecular and morphologic relapses in the study cohort (*n* = 303).

Between 01/01/2010 and 12/31/2019, 303 patients from nine centers with newly diagnosed CBF or *NPM1*-mutated AML (*RUNX1::RUNX1T1*, 19%; *CBFB::MYH11*, 27%; *NPM1*, 53%) who were in first complete remission and who had MRD monitoring after first-line intensive chemotherapy were included. In the 266 patients evaluated for *FLT3* mutational status, 40 had a *FLT3*-ITD mutation (all but two with concomitant *NPM1* mutation) and two had a *FLT3*-TKD mutation (one with concomitant *NPM1* mutation). Among included patients, 153 (51%) never relapsed (median follow-up of 1648 days after first CR; interquartile range [IQR]: 1101–1976), 95 (31%) had molecular relapse, with a median of 288 days (IQR: 229–384) from first CR, and 55 (18%) had upfront morphologic relapse (i.e., without prior molecular relapse detected), with a median of 351 days (IQR: 240–519; Fig. 1; Fig. 2). Among the 95 patients with molecular relapse, 53 (56%) received preemptive therapy (“preemptive” group with a median of 49 days [IQR: 27–75] from molecular relapse to treatment initiation) while 42 (44%) progressed to morphologic relapse by the time salvage therapy was initiated (“mol-morphologic relapse” group with a median of 62 days [IQR:38–132] from molecular relapse to treatment initiation at which point morphologic relapse was observed; Fig. 1). Although the cumulative incidence of morphologic relapse at three years was similar between patients with *RUNX1::RUNX1T1*, *CBFB::MYH11*, and *NPM1*-mutated AML (18% [8–28%], 16% [8–24%], and 19% [13–25%], respectively), the cumulative incidence of molecular relapse was lower in patients with *RUNX1::RUNX1T1* AML (23% [12–34%], 31% [21–41%], and 34% [27–42%], respectively); Supplementary Fig. 1). Molecular relapse occurred later in patients with *RUNX1::RUNX1T1* AML (median of 377 days [269–452] *vs*. 269 [199–371] *vs*. 279 [233–371]) while morphologic relapse occurred earlier in these patients (median of 288 [170–570] *vs*. 337 [305–456] *vs*. 355 [245–527]). There was a significant correlation between BM and PB MRD in patients for whom paired results were available, either after post-induction (*n* = 93; *P* < 0.001), at best response during first-line therapy (*n* = 161; *P* < 0.001), or at molecular relapse (*n* = 34; *P* < 0.001; Supplementary Fig. 2). With a quantification cut-off of 0.001%, 94 samples at best response were concordant whereas 64 samples were evaluated as negative in PB but positive in BM and 3 as positive in PB and negative in BM. Among the 34 paired samples available at molecular relapse, three were only detectable in BM whereas all others were detectable in PB and BM.

Patients with molecular and upfront morphologic relapse had higher white blood cell (WBC) count at diagnosis (12 *vs*. 22 *vs*. 30 G/l in those without relapse, molecular relapse, and morphological relapse, respectively, *P* = 0.003) and had lower MRD log-reduction in PB after induction therapy (4.15 *vs*. 3.71 *vs*. 3.64, respectively, *P* = 0.04). Best MRD response during first-line treatment was not different between the three groups. In addition, patients with upfront morphologic relapse were more likely to have CR with positive MRD other than CR_MRD-LL_ by the end of treatment in peripheral blood (2% *vs*. 16% *vs*. 32%, respectively, *P* < 0.001) and to have received less than three consolidation cycles (7% *vs*. 7% *vs*. 22%, respectively, *P* = 0.003; Table 1). Patients with any level of MRD in the bone marrow after first-line therapy were more likely to have molecular relapse whereas almost all patients with MRD other than CR_MRD-LL_ in peripheral blood (*n* = 22) eventually relapsed, either molecularly or morphologically (Supplementary Fig. 3). Similar results were observed when patients with CBF and *NPM1*-mutated AML were analyzed separately or when patients with 2022 ELN favorable AML (i.e., CBF and AML with *NPM1* mutations and without *FLT3*-ITD mutations) were considered (Supplementary Tables 1 to 3). However, more patients with *NPM1* and *FLT3*-ITD mutations experienced molecular and morphologic relapse (12% *vs*. 34% *vs*. 29%, respectively, *P* = 0.01, Supplementary Table 2). There was no difference in patient characteristics according to the type of relapse (“preemptive”, “mol-morphologic relapse”, and “upfront morphologic relapse” groups) besides less consolidation cycles received during first-line therapy in patients with upfront morphologic relapse (Supplementary Table 4).

**Table 1:** Clinical characteristics of study cohort (*n* = 303), stratified according to the type of relapse (no relapse *vs*. molecular relapse *vs*. upfront morphologic relapse).

Characteristic	All patients (*n* = 303)	No relapse (*n* = 153)	Molecular relapse (*n* = 95)	Morphologic relapse (*n* = 55)	*P*
Age at diagnosis, years	47 (37–53)	47 (35–52)	48 (38–53)	50 (39–57)	0.070
Female gender, *n* (%)	151 (50%)	86 (56%)	45 (47%)	20 (36%)	0.035
Leukocytes at diagnosis, G/l	16 (5–57)	12 (4–35)	22 (6–75)	30 (12–66)	0.003
Type of driver, *n* (%)					0.36
* t* (8;21)	59 (19%)	35 (23%)	14 (15%)	10 (18%)	
inv (16)	83 (27%)	45 (29%)	25 (26%)	13 (24%)	
* NPM1*mut	161 (53%)	73 (48%)	56 (59%)	32 (58%)	
Additional cytogenetic abnormalities, *n* (%)	81 (27%)	45 (30%)	23 (25%)	13 (24%)	0.50
Number of consolidation cycles, *n* (%)					0.003
1	6 (2%)	4 (3%)	1 (1%)	1 (2%)	
2	22 (7%)	7 (5%)	4 (4%)	11 (20%)	
3	269 (90%)	139 (93%)	87 (93%)	43 (78%)	
Post-induction ratio BM, log reduction	3.27 (2.61–4.11)	3.41 (2.73–4.32)	3.13 (2.58–3.68)	3.16 (2.55–3.85)	0.10
* Missing*	*94*	*42*	*23*	*29*	
Post-induction ratio PB, log reduction	3.89 (3.18–4.77)	4.15 (3.34–5.19)	3.71 (3.23–4.68)	3.64 (3.06–3.96)	0.040
* Missing*	*181*	*100*	*50*	*31*	
* Missing both BM and PB*	*63*	*36*	*15*	*12*	
Best ratio BM, log reduction	4.85 (3.91–5.81)	4.81 (3.95–5.72)	4.88 (3.91–5.81)	5.00 (3.21–5.99)	>0.99
* Missing*	*62*	*24*	*14*	*24*	
Best ratio PB, log reduction	5.35 (4.71–5.91)	5.35 (4.81–5.91)	5.41 (4.85–5.93)	5.11 (3.81–5.67)	0.31
* Missing*	*130*	*66*	*37*	*27*	
* Missing both BM and PB*	*37*	*20*	*8*	*9*	
Best type of CR in BM, *n* (%)					0.15
CR_MRD-_	105 (40%)	61 (44%)	30 (33%)	14 (40%)	
CR_MRD-LL_	66 (25%)	37 (27%)	24 (27%)	5 (14%)	
CR_MRD+_ other than CR_MRD-LL_	92 (35%)	40 (29%)	36 (40%)	16 (46%)	
* Missing*	*40*	*15*	*5*	*20*	
Best type of CR in PB, *n* (%)					<0.001
CR_MRD-_	148 (79%)	84 (89%)	47 (75%)	17 (55%)	
CR_MRD-LL_	18 (10%)	8 (9%)	6 (9%)	4 (13%)	
CR_MRD+_ other than CR_MRD-LL_	22 (12%)	2 (2%)	10 (16%)	10 (32%)	
* Missing*	*115*	*59*	*32*	*24*	

*BM* bone marrow, *CR* complete remission, *LL* low-level, *MRD* measurable residual disease, *PB* peripheral blood.

The best MRD ratio during first-line treatment was used to define best type of CR.

### Outcome of patients according to the type of relapse

**Fig. 3:**
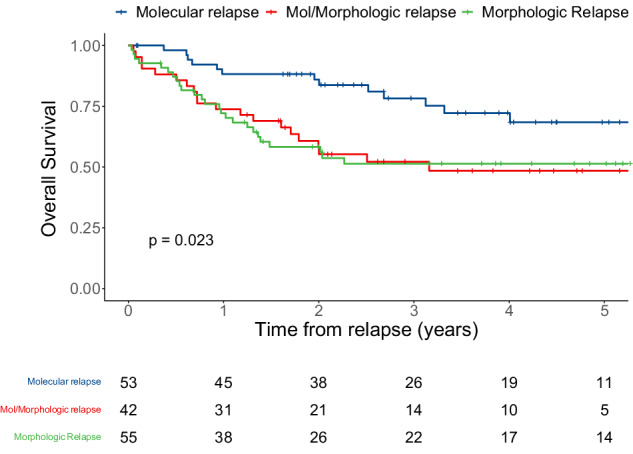
Overall survival of 150 patients with CBF or *NPM1*-mutated AML who relapsed during follow-up, stratified by the type of relapse (molecular relapse with preemptive therapy *vs*. molecular relapse with morphologic relapse at the time of salvage therapy *vs*. upfront morphologic relapse), calculated from the time of first detected relapse.

The multistate model and the number of patients entering the state transition matrix are described in Fig. 1A. During follow-up of the whole cohort, 50% of patients stayed alive and in remission (with only 3 deaths in patients who remained in remission). In patients who relapsed, predicted probabilities of being alive after preemptive therapy for molecular relapse or after morphologic relapse were 13 and 17%, respectively, whereas 4% died after preemptive therapy for molecular relapse and 15% after morphologic relapse (Fig. 1B). Patients who received preemptive therapy had a better OS than those who received salvage therapy after having progressed from molecular to morphologic relapse, and those who had upfront morphologic relapse (three-year OS of 78% *vs*. 52% *vs*. 51%, respectively, *P* = 0.02; Fig. 3), as when considering CBF and *NPM1*-mutated AML patient separately (Supplementary Fig. 4). Similar results were observed when calculating survival probabilities from the day of starting salvage therapy after excluding two patients who declined salvage therapy, rather than on the day of first detected relapse (Supplementary Fig. 5). Interestingly, treatment-related mortality was significantly higher in patients who received salvage therapy while in morphologic relapse (*P* = 0.015; Supplementary Fig. 6).

To study the relationship between the type of relapse and outcomes in more detail, we evaluated univariable and multivariable regression models for the endpoint of OS calculated from the time of first detected relapse. In univariable analysis, age at diagnosis (hazard ratio [HR] = 1.03 [1.01–1.06], *P* = 0.022), number of consolidation cycles (HR = 0.59 [0.35–0.99], *P* = 0.046), best MRD log reduction in bone marrow (HR = 0.66 [0.51–0.85], *P* = 0.001), CR with MRD other than at low-level in bone marrow and in peripheral blood at the end of first-line therapy (HR = 2.20 [1.08–4.50], *P* = 0.03, and HR = 2.25 [1.07–4.73], *P* = 0.033, respectively), and both “mol-morphologic” and upfront morphologic relapses (HR = 2.34 [1.16–4.71], *P* = 0.017, and HR = 2.31 [1.18–4.51], *P* = 0.015) were associated with OS (Table 2). After multivariable adjustment, excluding potential colinear variables, best MRD log reduction in bone marrow (HR = 0.71 [0.55–0.93], *P* = 0.011) and “mol-morphologic” relapse (HR = 2.17 [1.00–4.71], *P* = 0.05) were independently associated with OS.

**Table 2:** Univariable and multivariable regression models of patients with relapse (*n* = 150) for overall survival.

	HR (95% CI)	*P*	HR (95% CI)	*P*
Age at Diagnosis	1.03 (1.00–1.06)	0.022	1.02 (0.99–1.06)	0.2
Female Gender	1.12 (0.67–1.88)	0.7		
Leukocytes at Diagnosis	1.00 (1.00–1.01)	0.5		
Type of Driver				
*t* (8;21)	Ref.			
inv(16)	0.86 (0.37–1.95)	0.7		
* NPM1*mut	1.04 (0.51–2.10)	>0.9		
Additional cytogenetic abnormalities	1.20 (0.68–2.14)	0.5		
* FLT3*-ITD mutation	1.00 (0.53–1.89)	>0.9		
Number of consolidation cycles	0.59 (0.35–0.99)	0.046	0.63 (0.28–1.42)	0.14
Post–induction ratio BM, log reduction	1.01 (0.74–1.38)	>0.9		
Post–induction ratio PB, log reduction	1.03 (0.73–1.46)	0.9		
Best ratio BM, log reduction	0.66 (0.51–0.85)	0.001	0.71 (0.55–0.93)	0.011
Best ratio PB, log reduction	0.81 (0.63–1.04)	0.10		
Best type of CR in BM				
CR_MRD-_	Ref.			
CR_MRD-LL_	1.83 (0.81–4.15)	0.15		
CR_MRD+_ other than CR_MRD-LL_	2.20 (1.08–4.50)	0.03		
Best type of CR in PB				
CR_MRD-_	Ref.			
CR_MRD-LL_	2.36 (0.88–6.32)	0.089		
CR_MRD+_ other than CR_MRD-LL_	2.25 (1.07–4.73)	0.033		
Relapse time	0.56 (0.33–0.97)	0.038	0.70 (0.37–1.34)	0.3
Type of relapse				
Molecular relapse – preemptive therapy	Ref.		Ref.	
Molecular–morphologic relapse	2.34 (1.16–4.71)	0.017	2.17 (1.00–4.71)	0.05
Upfront morphologic relapse	2.31 (1.18–4.51)	0.015	1.99 (0.85–4.65)	0.11

*BM* bone marrow, *CI* confidence interval, *CR* complete remission, *HR* hazard ratio, *LL* low-level, *MRD* measurable residual disease, *PB* peripheral blood.

### Salvage therapy

The type of salvage therapy for the “preemptive”, “mol-morphologic relapse”, and “upfront morphologic relapse” groups are described in Table 3. Time from relapse detection to salvage therapy initiation was significantly shorter in patients with upfront morphologic relapse (*P* < 0.001). Although not statistically different, patients with molecular relapse who received preemptive therapy while in molecular relapse had a shorter time to treatment than patients who have progressed to morphologic relapse at treatment initiation (49 *vs*. 62 days; *P* = 0.08). Also, significantly more patients in the “preemptive” group received upfront allogeneic HCT (19 [36%] *vs*. 2 [5%] *vs*. 2 [4%] in the “preemptive”, “mol-morphologic relapse”, and “upfront morphologic relapse” groups, respectively).

**Table 3:** Type of salvage therapy in 150 patients with CBF and *NPM1*-mutated AML patients with relapse, stratified by the type of relapse (molecular relapse with preemptive therapy *vs*. molecular relapse with morphologic relapse at the time of salvage therapy *vs*. upfront morphologic relapse).

Characteristic	All relapses (*n* = 150)	Molecular relapse (*n* = 53)	Molecular-morphologic relapse (*n* = 42)	Upfront morphologic relapse (*n* = 55)
Time from relapse to salvage therapy (IQR), days	33 (14–64)	49 (27–75)	62 (38–132)	10 (4–22)
Type of salvage treatment, *n* (%)				
* Upfront allogeneic HCT*	23 (15%)	19 (36%)	2 (5%)	2 (4%)
* Intensive chemotherapy*	95 (63%)	21 (40%)	33 (79%)	41 (75%)
GO-containing chemotherapy	34 (23%)	10 (19%)	11 (26%)	13 (24%)
* Non-intensive chemotherapy*	30 (20%)	13 (25%)	5 (12%)	12 (22%)
IDH inhibitors	3 (2%)	2 (4%)	0	1 (2%)
FLT3 inhibitors	8 (5%)	2 (4%)	2 (5%)	4 (7%)
Azacitidine	3 (2%)	1 (2%)	0	2 (4%)
Azacitidine-GO	4 (3%)	4 (8%)	0	0
Azacitidine-venetoclax	5 (3%)	2 (4%)	2 (5%)	1 (2%)
GO	5 (3%)	0	1 (2%)	4 (7%)
Other	2 (1%)	2 (4%)	0	0
* No treatment*	2 (1%)	0	2 (5%)	0
Allogeneic HCT, *n* (%)	121 (81%)	45 (85%)	31 (74%)	45 (82%)
* Sequential allogeneic HCT*	29 (19%)	12 (23%)	8 (19%)	9 (16%)

*IQR* interquartile range, *GO* gemtuzumab ozogamicin, *HCT* hematopoietic cell transplantation.

Salvage treatment with intensive chemotherapy was also less common in the “preemptive” group (21 [40%] *vs*. 33 [79%] *vs*. 41 [75%], respectively; *P* < 0.001). Among the 19 patients in the “preemptive” group receiving upfront allogeneic HCT, 11 of them received transplant from a HLA-matched sibling donor and six received a sequential conditioning regimen. The rate of allogeneic HCT was similar between the three groups (85% *vs*. 74% *vs*. 82%, *P* = 0.38). Among the 28 patients who did not undergo transplant (19%), only 7 were still alive at three years (*RUNX1::RUNX1T1* [*n* = 1], *CBFB::MYH11* [*n* = 4], and *NPM1* mutation [*n* = 2]; molecular relapse [*n* = 6], morphologic relapse [*n* = 1]; Supplementary Fig. 7).

**Fig. 4:**
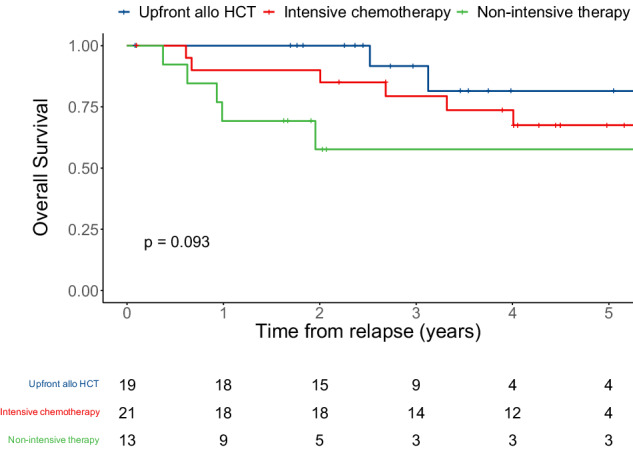
Overall survival of 53 patients with CBF or *NPM1*-mutated AML with molecular relapse who received preemptive therapy, stratified by the type of salvage therapy (upfront allogeneic hematopoietic cell transplantation (HCT) *vs*. intensive chemotherapy *vs*. non-intensive chemotherapy), calculated from the time of first detected relapse.

Specific results of each category of preemptive therapy (upfront allogeneic HCT [*n* = 19], intensive chemotherapy [*n* = 21], and non-intensive therapy [*n* = 13]) are summarized in Supplementary Table 5. In the 19 patients who proceeded to upfront allogeneic HCT, 15 (79%) achieved complete molecular remission after allogeneic HCT with only two relapsing after allogeneic HCT. More patients achieved complete molecular remission after intensive chemotherapy in comparison to patients who received non-intensive chemotherapies (11 [52%] *vs*. 2 [15%]) and had lower levels of transcript before allogeneic HCT (0.003 [0.001–0.29] *vs*. 2.5 [0.01–11.3]). Different OS rates, that did not reach statistical significance, were observed between the three treatment strategies (92% *vs*. 79% *vs*. 58% at three years for upfront allogeneic HCT, intensive chemotherapy, and less intensive therapy, respectively, *P* = 0.09; Fig. 4). Two-by-two comparisons showed that there was a significant difference in OS between patients receiving upfront allogeneic HCT and non-intensive chemotherapy (*P* = 0.027) whereas OS of patients receiving upfront allogeneic HCT and intensive chemotherapy (*P* = 0.36) and OS of patients receiving intensive and non-intensive chemotherapy were not different (*P* = 0.24).

## Discussion

In this study, we evaluated the outcome of CBF and *NPM1*-mutated AML patients who were monitored for molecular MRD after first CR following intensive induction and consolidation therapy. Relapse was observed in 50% of patients with two thirds of these relapses detected at the molecular level. Among patients with molecular relapse, preemptive therapy could be initiated in half of them before overt morphologic relapse. These preemptively treated patients had a better OS than those treated with active disease. Preemptive therapy was very heterogeneous which reflects the lack of standardization regarding the optimal strategy for molecular relapse.

Because detection of MRD during standard therapy (i.e., after induction and consolidation therapy, before allogeneic HCT) is associated with increased relapse rates and decreased survival in AML patients, it is now fully integrated into the routine management of these patients [25, 26]. Recent international guidelines also consider molecular monitoring of patients with CBF and *NPM1*-mutated AML beyond first-line therapy [15]. Although molecular monitoring can detect impending morphologic relapse, it is not clear if there is sufficient time to intervene before overt morphologic relapse and if early treatment can benefit these patients [19, 27]. As in previous studies [17, 19], we show that molecular monitoring is efficient in detecting relapse in CBF and *NPM1*-mutated patients. Despite the lack of standardization in MRD monitoring in our cohort, we could detect molecular relapse in two thirds of patients who relapse which compares favorably with previous studies showing that 60 to 73% of relapses could be detected at the molecular level in these patients [17, 19]. Similar to a previous study evaluating MRD molecular monitoring in CBF patients, preemptive therapy could be initiated before overt morphologic relapse in 35% of patients (42% in the previous study) [19]. Half of the patients with molecular relapse experienced rapid morphologic relapse (median of 58 days from first detection of molecular relapse to treatment initiation) which may advocate for immediate initiation of preemptive therapy at the first sign of molecular relapse. In addition, a significant number of patients relapsed morphologically without a previous detection of MRD by qPCR. It is possible that routine molecular monitoring every 3 months is inadequate in these patients (i.e., those with higher WBC count or with CR MRD+ other than CRMRD-LL after induction), and that the ELN recommendations for monitoring MRD every 4–6 weeks in blood could be more appropriate.

Due to potential heterogeneity in our cohort, we led subgroup analyses in CBF and *NPM1*-mutated AML patients. Although patients with *NPM1*-mutated AML were more likely to relapse (55% *vs*. 43% of relapses in patients with CBF-mutated AML), a similar proportion of these relapses were detected at the molecular level (64 and 62% of relapses, respectively). The cumulative incidence of molecular relapse was however lower in patients with *RUNX1::RUNX1T1* AML and occurred slightly later during follow-up. The outcome of patients following molecular relapse and preemptive therapy was similar in the different patient subsets. Similar results were observed when focusing on patients with 2022 ELN favorable AML. The terminology “favorable-risk AML”, must be used with caution as up to 45% of these patients will relapse and require salvage therapy, including allogeneic HCT. Whereas almost all patients with measurable MRD in both PB and BM eventually relapsed, not all patients with measurable MRD only in BM did. One may therefore ask whether patients with detectable MRD in both PB and BM at the end of first-line therapy should receive immediate salvage therapy, as their risk of relapse is very high, or at least have very close MRD monitoring.

Although only 18% of patients in the whole cohort had a change in management after molecular monitoring, these patients had improved outcome in comparison to other relapse patients (three-year OS of 78% *vs*. 51% for those receiving therapy with active disease). As in a previous report where 10% of molecularly monitored CBF patients could receive preemptive therapy [19], monitoring all patients would only be of interest if that subgroup can benefit from earlier therapeutic intervention. In contrast to one study that compared MRD, as assessed by flow cytometry, and morphologic relapse [27], and similarly to one study that compared molecular failure and morphologic relapse [17], we showed that patients who received salvage therapy while in molecular relapse, but persistent morphologic remission, had better outcomes. Our data show that patients who received preemptive salvage therapy had significantly lower treatment-related mortality than patients who were treated at morphologic relapse. Whether preemptively treated patients would have had a less favorable outcome had they received salvage therapy at the time of morphologic relapse is however unknown. We cannot exclude a potential bias when comparing outcome of patients with molecular relapse who received preemptive therapy as compared to those receiving salvage therapy after having progressed from molecular to morphologic relapse. Patients with rapidly progressing disease (i.e., those having progressed from molecular to morphologic relapse at treatment initiation) might also have more aggressive disease that may be less likely to respond to therapy and thus have decreased survival. Our data show however that patients in the “mol-morphologic” group did not have more rapidly relapsing disease as time to treatment initiation was longer in these patients and thus decreased outcome in these patients might rather be to slower initiation of salvage therapy.

We also explored the outcomes of preemptively treated patients according to the type of salvage therapy. Interestingly, with similar levels of CBF transcripts or *NPMI* mutations at the time of treatment initiation, patients receiving upfront allogeneic HCT had favorable outcomes. Although, it did not reach statistical significance, patients receiving intensive salvage therapy had better outcomes than patients receiving non-intensive therapies. The proportion of patients receiving allogeneic HCT was similar in these two groups, but MRD reduction was more important in those receiving intensive chemotherapy, and we can speculate that lowering MRD levels before HCT can be helpful in those for which upfront allogeneic HCT is not feasible. Due to small numbers and heterogeneity in treatment, we could not however evaluate each individual non-intensive therapy although azacitidine monotherapy [16], azacitidine and venetoclax combination [18], and FLT3 inhibitors [28] have shown some promise in the context of molecular relapse. It should be noted that venetoclax, FLT3 and IDH inhibitors were not fully available during the study period in France. It is anticipated that these treatments could be a potent strategy likely less toxic than intensive chemotherapy in this situation [29].

Several limitations of our study must be acknowledged. First, due to the retrospective nature of our study and the non-randomized allocation of patient to different treatment strategies at relapse, we cannot draw definitive conclusions regarding management of patients with molecular relapse. Also, because MRD monitoring was not randomized, we cannot confirm that MRD monitoring, and prompt treatment initiation can benefit patients, although one unpublished prospective randomized study showed that patients with *NPM1* and *FLT3*-ITD mutations had improved OS if they were monitored molecularly (*Potter* et al. *EHA annual meeting 2023*). Second, there were missing data regarding evaluation of MRD during first-line treatment and we should therefore be cautious when interpreting its association with outcome. Third, the generalizability of our results may be limited as we could not identify patients who did not have molecular follow-up. Additionally, as patients included in this study were younger and fit to receive allogeneic HCT (81% of all relapse patients), these results might not be applicable to patients not fit for transplant. Fourth, molecular follow-up was not standardized, including reagents and equipment, frequency of molecular monitoring and source of samples (PB and/or BM). Although, PB and BM sampling at the time of molecular relapse seem to give similar results, because the frequency of MRD monitoring was not standardized, our data does not modify the current recommendation to perform MRD monitoring every 4 to 6 weeks in PB and/or every 3 months in BM in these patients [15]. Fifth, we could not evaluate other techniques for MRD monitoring such as flow cytometry. Sixth, salvage therapy was very heterogeneous with a small proportion of patients receiving each type of salvage therapy precluding any definitive conclusions on the type of preemptive therapy that can be recommended.

Patients treated during molecular relapse had a better OS than those treated with active disease. Although these results need to be confirmed in larger studies, it supports molecular monitoring during follow-up to start preemptive therapy before overt morphologic relapse and validates the value of the new 2022 ELN endpoints (i.e., EFS-MRD, RFS-MRD, CIR-MRD) for a better description of clinical trial results. In addition, similar data with follow-up of MRD using other techniques such as flow cytometry and next-generation sequencing are urgently needed for the management of most AML patients who cannot be followed by qPCR.

### Supplementary information

Supplemental material

## Data Availability

The dataset analyzed in this study is available from the corresponding author on reasonable request.
