# Supplementary material for: Molecular relapse after first-line intensive therapy in patients with CBF or NPM1-mutated acute myeloid leukemia – a FILO study
Source: Leukemia. 2024 Jul 17;38(9):1949–57. doi: 10.1038/s41375-024-02335-2 (PMC11347360; doi:10.1038/s41375-024-02335-2)

**Molecular Relapse after First-Line Intensive Therapy in Patients with Core-Binding Factor or *NPM1*-mutated Acute Myeloid Leukemia**

Corentin Orvain, Sarah Bertoli, Pierre Peterlin, Yohann Desbrosses, Pierre-Yves Dumas, Alexandre Iat, Marie-Anne Hospital, Martin Carre, Emmanuelle Tavernier, Anne Bouvier, Audrey Bidet, Sylvie Tondeur, Florian Renosi, Marie-Joelle Mozziconacci, Pascale Flandrin-Gresta, Bérengère Dadone-Montaudié, Eric Delabesse, Arnaud Pigneux, Mathilde Hunault-Berger, and Christian Recher on behalf of the French Innovative Leukemia Organization (FILO)

**Supplementary Data**

**Supplementary Table 1.** Clinical characteristics of patients with CBF AML (n=142), stratified according to relapse status (no relapse *vs.* molecular relapse *vs.* upfront morphologic relapse).

| Characteristic | All patients (n=142) | No relapse (n=80) | Molecular Relapse (n=39) | Morphologic Relapse (n=23) | *P* |
| --- | --- | --- | --- | --- | --- |
| Age at diagnosis, years | 44 (33-52) | 44 (33-51) | 44 (33-53) | 42 (36-54) | 0.73 |
| Female gender, n (%) | 57 (40%) | 36 (45%) | 14 (36%) | 7 (30%) | 0.37 |
| Leukocytes at diagnosis, G/l | 14 (5-56) | 13 (5-51) | 12 (5-69) | 31 (16-64) | 0.12 |
| Type of driver, n (%) |  |  |  |  | 0.70 |
| t(8;21) | 59 (42%) | 35 (44%) | 14 (36%) | 10 (43%) |  |
| inv(16) | 83 (58%) | 45 (56%) | 25 (64%) | 13 (57%) |  |
| Additional cytogenetic abnormalities, n (%) | 68 (51%) | 40 (52%) | 19 (51%) | 9 (47%) | 0.94 |
| Number of consolidation cycles, n (%) |  |  |  |  | 0.075 |
| 1 | 3 (2%) | 2 (3%) | 1 (3%) | 0 |  |
| 2 | 12 (9%) | 3 (4%) | 4 (10%) | 5 (22%) |  |
| 3 | 126 (89%) | 74 (94%) | 34 (87%) | 18 (78%) |  |
| Post-induction ratio BM, log reduction | 2.95 (2.42-3.57) | 3.12 (2.43-3.70) | 2.82 (2.55-3.28) | 2.67 (2.07-3.13) | 0.17 |
| *Missing* | 37 | 14 | 11 | 12 |  |
| Post-induction ratio PB, log reduction | 3.60 (3.08-4.22) | 3.85 (3.09-4.64) | 3.50 (2.91-4.23) | 3.46 (3.10-3.81) | 0.44 |
| *Missing* | 89 | 51 | 25 | 13 |  |
| Best ratio BM, log reduction | 4.25 (3.54-4.87) | 4.41 (3.70-5.01) | 4.16 (3.60-4.79) | 3.21 (2.64-4.36) | 0.040 |
| *Missing* | 24 | 5 | 8 | 11 |  |
| Best ratio PB, log reduction | 5.00 (4.64-5.46) | 4.97 (4.67-5.45) | 5.03 (4.78-5.29) | 4.85 (3.49-5.80) | 0.78 |
| *Missing* | 59 | 31 | 17 | 11 |  |
| Best type of CR in BM, n (%) |  |  |  |  | 0.023 |
| CR_MRD-_ | 41 (32%) | 30 (39%) | 9 (24%) | 2 (14%) |  |
| CR_MRD-LL_ | 31 (24%) | 21 (27%) | 9 (24%) | 1 (7%) |  |
| CR_MRD+_ other than CR_MRD-LL_ | 56 (44%) | 26 (34%) | 19 (51%) | 11 (79%) |  |
| *Missing* | 14 | 3 | 2 | 9 |  |
| Best type of CR in PB, n (%) |  |  |  |  | 0.004 |
| CR_MRD-_ | 72 (80%) | 46 (90%) | 18 (75%) | 8 (53%) |  |
| CR_MRD-LL_ | 9 (10%) | 4 (8%) | 3 (12%) | 2 (13%) |  |
| CR_MRD+_ other than CR_MRD-LL_ | 9 (10%) | 1 (2%) | 3 (12%) | 5 (33%) |  |
| *Missing* | 52 | 29 | 15 | 8 |  |

**Abbreviations:** BM, bone marrow; CR, complete remission; LL, low-level; MRD, measurable residual disease; PB, peripheral blood.

**Supplementary Table 2.** Clinical characteristics of patients with *NPM1*-mutated AML (n=161), stratified according to relapse status (no relapse *vs.* molecular relapse *vs.* upfront morphologic relapse).

| Characteristic | All patients (n=161) | No relapse (n=73) | Molecular Relapse (n=56) | Morphologic Relapse (n=32) | *P* |
| --- | --- | --- | --- | --- | --- |
| Age at diagnosis, years | 49 (40-55) | 49 (37-54) | 48 (42-53) | 54 (44-58) | 0.087 |
| Female gender, n (%) | 94 (58%) | 50 (68%) | 31 (55%) | 13 (41%) | 0.024 |
| Leukocytes at diagnosis, G/l | 17 (5-57) | 9 (4-26) | 25 (8-77) | 29 (8-68) | 0.010 |
| Additional cytogenetic abnormalities, n (%) | 12 (8%) | 5 (7%) | 4 (7%) | 3 (9%) | 0.86 |
| *FLT3*-ITD mutation, n (%) | 37 (23%) | 9 (12%) | 19 (34%) | 9 (29%) | 0.011 |
| Number of consolidation cycles, n (%) |  |  |  |  | 0.002 |
| 1 | 3 (2%) | 2 (3%) | 0 | 1 (3%) |  |
| 2 | 10 (6%) | 4 (5%) | 0 | 6 (19%) |  |
| 3 | 144 (92%) | 65 (92%) | 55 (100%) | 25 (78%) |  |
| Post-induction ratio BM, log reduction | 3.66 (2.96-4.64) | 4.13 (3.27-4.81) | 3.34 (2.70-3.85) | 3.41 (2.96-4.86) | 0.010 |
| *Missing* | 57 | 28 | 12 | 17 |  |
| Post-induction ratio PB, log reduction | 4.15 (3.48-5.12) | 4.91 (4.41-5.96) | 3.93 (3.35-4.73) | 3.71 (3.05-4.32) | 0.007 |
| *Missing* | 92 | 49 | 25 | 18 |  |
| Best ratio BM, log reduction | 5.73 (4.81-6.08) | 5.74 (4.81-6.13) | 5.50 (4.45-6.02) | 5.93 (5.15-6.17) | 0.34 |
| *Missing* | 38 | 19 | 6 | 13 |  |
| Best ratio PB, log reduction | 5.79 (4.96-6.11) | 5.87 (5.15-6.17) | 5.80 (4.99-6.11) | 5.40 (4.17-5.64) | 0.034 |
| *Missing* | 71 | 35 | 20 | 16 |  |
| Best type of CR in BM, n (%) |  |  |  |  | 0.61 |
| CR_MRD-_ | 64 (47%) | 31 (51%) | 21 (40%) | 12 (57%) |  |
| CR_MRD-LL_ | 35 (26%) | 16 (26%) | 15 (28%) | 4 (19%) |  |
| CR_MRD+_ other than CR_MRD-LL_ | 36 (27%) | 14 (23%) | 17 (32%) | 5 (24%) |  |
| *Missing* | 26 | 12 | 3 | 11 |  |
| Best type of CR in PB, n (%) |  |  |  |  | 0.016 |
| CR_MRD-_ | 76 (78%) | 38 (88%) | 29 (74%) | 9 (56%) |  |
| CR_MRD-LL_ | 9 (9.2%) | 4 (9.3%) | 3 (7.7%) | 2 (12%) |  |
| CR_MRD+_ other than CR_MRD-LL_ | 13 (13%) | 1 (2.3%) | 7 (18%) | 5 (31%) |  |
| *Missing* | 63 | 30 | 17 | 16 |  |

**Abbreviations:** BM, bone marrow; CR, complete remission; LL, low-level; MRD, measurable residual disease; PB, peripheral blood.

**Supplementary Table 3.** Clinical characteristics of patients with 2022 ELN favorable AML (n=262), stratified according to relapse status (no relapse *vs.* molecular relapse *vs.* upfront morphologic relapse).

| Characteristic | All patients (n=262) | No relapse (n=144) | Molecular Relapse (n=74) | Morphologic Relapse (n=44) | *P* |
| --- | --- | --- | --- | --- | --- |
| Age at Diagnosis, years | 47 (36-53) | 46 (35-52) | 48 (38-53) | 46 (37-57) | 0.39 |
| Female Gender, n (%) | 136 (52%) | 66 (46%) | 40 (54%) | 30 (68%) | 0.031 |
| Leukocytes at Diagnosis, G/l | 14 (5-53) | 12 (4-40) | 14 (5-67) | 30 (16-74) | 0.009 |
| Type of Driver, n (%) |  |  |  |  | 0.92 |
| t(8;21) | 59 (23%) | 35 (24%) | 14 (19%) | 10 (23%) |  |
| inv(16) | 83 (32%) | 45 (31%) | 25 (34%) | 13 (30%) |  |
| *NPM1*mut | 120 (46%) | 64 (44%) | 35 (47%) | 21 (48%) |  |
| Additional cytogenetic abnormalities, n (%) | 77 (31%) | 44 (32%) | 21 (29%) | 12 (30%) | 0.93 |
| Number of Consolidation Cycles, n (%) |  |  |  |  | 0.019 |
| 1 | 3 (1%) | 2 (1%) | 1 (1%) | 0 |  |
| 2 | 20 (8%) | 7 (5%) | 4 (5%) | 9 (20%) |  |
| 3 | 236 (91%) | 132 (94%) | 69 (93%) | 35 (80%) |  |
| Post-Induction Ratio BM, log reduction | 3.31 (2.64-4.11) | 3.44 (2.68-4.21) | 3.19 (2.61-3.74) | 3.30 (2.43-3.76) | 0.28 |
| *Missing* | *84* | *40* | *20* | *24* |  |
| Post-Induction Ratio PB, log reduction | 3.94 (3.16-4.71) | 4.13 (3.27-4.96) | 3.93 (3.27-4.70) | 3.39 (2.98-3.89) | 0.043 |
| *Missing* | *165* | *98* | *42* | *25* |  |
| Best Ratio BM, log reduction | 4.81 (3.83-5.72) | 4.81 (3.91-5.71) | 4.85 (3.79-5.43) | 4.92 (3.15-5.95) | 0.87 |
| *Missing* | *54* | *22* | *13* | *19* |  |
| Best Ratio PB, log reduction | 5.15 (4.68-5.85) | 5.22 (4.74-5.87) | 5.23 (4.79-5.91) | 5.10 (3.84-5.65) | 0.40 |
| *Missing* | *120* | *64* | *33* | *23* |  |
| Best Type of CR in BM, n (%) |  |  |  |  | 0.14 |
| CR MRD- | 88 (38%) | 57 (44%) | 21 (30%) | 10 (34%) |  |
| CR MRD-LL | 60 (26%) | 35 (27%) | 20 (29%) | 5 (17%) |  |
| CR MRD+ | 81 (35%) | 38 (29%) | 29 (41%) | 14 (48%) |  |
| *Missing* | *33* | *14* | *4* | *15* |  |
| Best Type of CR in PB, n (%) |  |  |  |  | <0.001 |
| CR MRD- | 128 (82%) | 78 (91%) | 36 (78%) | 14 (58%) |  |
| CR MRD-LL | 11 (7%) | 6 (7%) | 3 (7%) | 2 (8%) |  |
| CR MRD+ | 17 (11%) | 2 (2%) | 7 (15%) | 8 (33%) |  |
| *Missing* | *106* | *58* | *28* | *20* |  |

**Supplementary Table 4.** Clinical characteristics of patients with relapse (n=150), stratified by the type of relapse (molecular relapse with preemptive therapy *vs.* molecular relapse with morphologic relapse at the time of salvage therapy *vs.* upfront morphologic relapse).

| Characteristic | All relapses (n=150) | Molecular relapse (n=53) | Mol-morphologic relapse (n=42) | Upfront morphologic relapse (n=55) | *P* |
| --- | --- | --- | --- | --- | --- |
| Age at diagnosis, years | 48 (39-55) | 44 (38-53) | 49 (41-53) | 50 (39-57) | 0.25 |
| Female gender, n (%) | 65 (43%) | 30 (57%) | 15 (36%) | 20 (36%) | 0.053 |
| Leukocytes at diagnosis, G/l | 26 (7-71) | 18 (6-68) | 25 (7-78) | 30 (12-66) | 0.84 |
| Type of driver, n (%) |  |  |  |  | 0.86 |
| t(8;21) | 24 (16%) | 9 (17%) | 5 (12%) | 10 (18%) |  |
| inv(16) | 38 (25%) | 15 (28%) | 10 (24%) | 13 (24%) |  |
| *NPM1*mut | 88 (59%) | 29 (55%) | 27 (64%) | 32 (58%) |  |
| Additional cytogenetic abnormalities, n (%) | 35 (24%) | 14 (27%) | 9 (22%) | 12 (24%) | 0.85 |
| Number of consolidation cycles, n (%) |  |  |  |  | 0.023 |
| 1 | 2 (1%) | 1 (2%) | 0 | 1 (2%) |  |
| 2 | 15 (10%) | 3 (6%) | 1 (2%) | 11 (20%) |  |
| 3 | 132 (88%) | 49 (93%) | 40 (97%) | 43 (78%) |  |
| Post-induction ratio BM, log reduction | 3.13 (2.58-3.74) | 3.21 (2.57-3.56) | 2.95 (2.67-3.75) | 3.16 (2.55-3.85) | >0.99 |
| *Missing* | 52 | 9 | 14 | 29 |  |
| Post-induction ratio PB, log reduction | 3.70 (3.16-4.44) | 3.94 (3.43-5.12) | 3.61 (2.92-4.21) | 3.64 (3.06-3.96) | 0.22 |
| *Missing* | 81 | 28 | 22 | 31 |  |
| Best ratio BM, log reduction | 4.89 (3.80-5.90) | 4.65 (3.79-5.72) | 4.98 (4.12-5.83) | 5.00 (3.21-5.99) | 0.92 |
| *Missing* | 38 | 7 | 7 | 24 |  |
| Best ratio PB, log reduction | 5.33 (4.62-5.90) | 5.47 (4.93-5.91) | 5.31 (4.56-6.00) | 5.11 (3.81-5.67) | 0.43 |
| *Missing* | 64 | 20 | 17 | 27 |  |
| Best type of CR in BM, n (%) |  |  |  |  | 0.29 |
| CR_MRD-_ | 44 (35%) | 18 (36%) | 12 (30%) | 14 (40%) |  |
| CR_MRD-LL_ | 29 (23%) | 10 (20%) | 14 (35%) | 5 (14%) |  |
| CR_MRD+_ other than CR_MRD-LL_ | 52 (42%) | 22 (44%) | 14 (35%) | 16 (46%) |  |
| *Missing* | 25 | 3 | 2 | 20 |  |
| Best type of CR in PB, n (%) |  |  |  |  | 0.23 |
| CR_MRD-_ | 64 (68%) | 29 (78%) | 18 (69%) | 17 (55%) |  |
| CR_MRD-LL_ | 10 (11%) | 4 (11%) | 2 (7.7%) | 4 (13%) |  |
| CR_MRD+_ other than CR_MRD-LL_ | 20 (21%) | 4 (11%) | 6 (23%) | 10 (32%) |  |
| *Missing* | 56 | 16 | 16 | 24 |  |

**Abbreviations:** BM, bone marrow; CR, complete remission; LL, low-level; MRD, measurable residual disease; PB, peripheral blood.

**Supplementary Table 5.** Results of salvage therapy in patients with CBF and *NPM1*-mutated AML who received preemptive therapy at molecular relapse (n=53), stratified by type of salvage therapy (upfront allogeneic hematopoietic cell transplantation vs. intensive chemotherapy vs. non-intensive chemotherapy).

| Characteristic | Upfront allogeneic HCT (n=19) | Intensive chemotherapy (n=21) | Non-intensive chemotherapy (n=13) |
| --- | --- | --- | --- |
| Time from relapse to treatment (IQR), days | 68 (52-105) | 41 (26-54) | 42 (17-65) |
| Age at salvage (IQR), years | 52 (39-56) | 41 (38-53) | 48 (43-54) |
| Level of transcript before salvage (IQR) | 1.6 (1.1-16.7) | 1.4 (0.3-17.8) | 6.0 (1.8-15) |
| Response after salvage, n (%) |  |  |  |
| CR_MRD-_ |  | 11 (52%) | 2 (15%) |
| CR_MRD-LL_ |  | 2 (10%) | 0 |
| CR_MRD+_ other than CR_MRD-LL_ |  | 6 (29%) | 5 (38%) |
| Allogeneic HCT, n (%) | 19 (100%) | 15 (71%) | 11 (85%) |
| Level of transcript before allogeneic HCT (IQR) | 1.6 (1.1-16.7) | 0.003 (0.001-0.29) | 2.5 (0.01-11.3) |
| Response after allogeneic HCT, n (%) |  |  |  |
| CR_MRD-_ | 15 (79%) | 10 (67%) | 5 (45%) |
| CR_MRD-LL_ | 1 (5%) | 1 (7%) | 2 (18%) |
| CR_MRD+_ other than CR_MRD-LL_ | 1 (5%) | 1 (7%) | 1 (9%) |
| Patients who proceeded to allogeneic HCT, n (%) | 19 (100%) | 15 (71%) | 11 (85%) |
| Molecular relapse | 2 (11%) | 3 (20%) | 1 (9%) |
| Morphologic relapse | 1 (5%) | 1 (7%) | 0 |

**Abbreviations:** CR, complete remission; HCT, hematopoietic cell transplantation; IQR, interquartile range; LL, low-level; MRD, measurable residual disease.

**Supplementary Figure 1.** Cumulative incidence of molecular and morphologic relapses in patient with *RUNX1-RUNX1T1* (n=59), *CBFB-MYH1* (n=83), and *NPM1*-mutated AML (n=161).

**
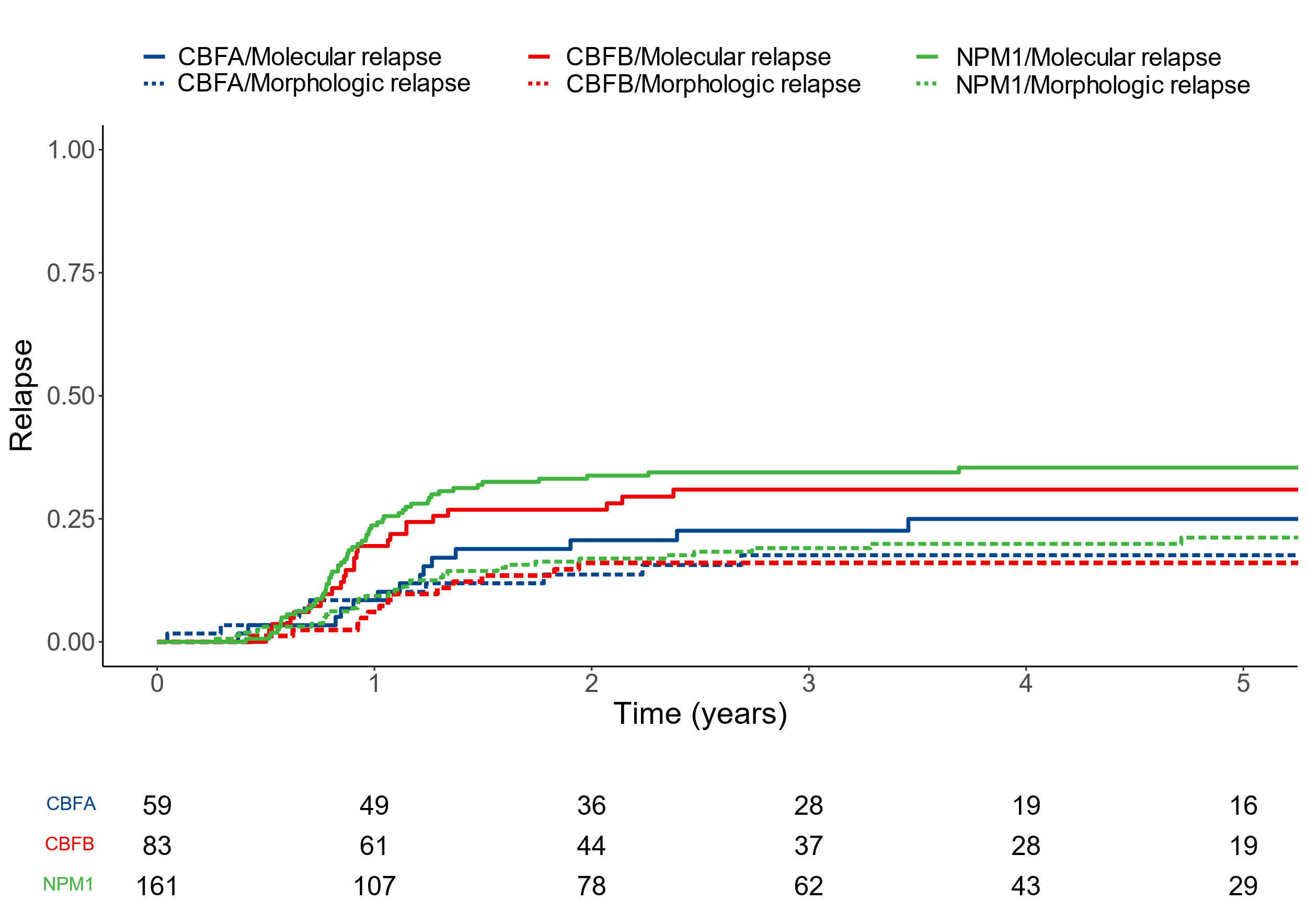
**

**Supplementary Figure 2.** Bone marrow (BM) and peripheral (PB) paired MRD samples (A) after induction therapy (n=93), (B) at best response (n=161), and (C) at molecular relapse (n=34).

**A.**

**
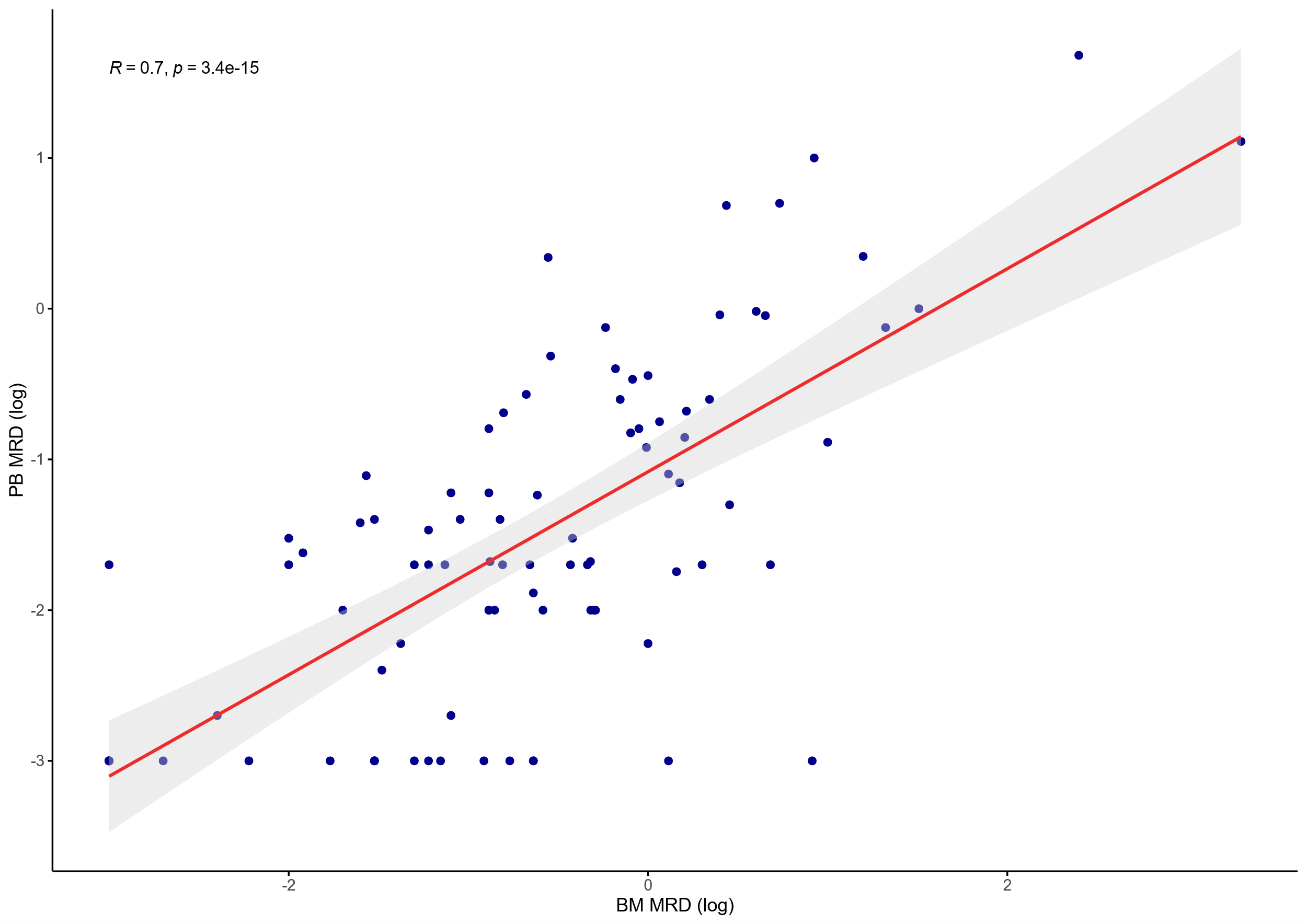
**

**B.**

**
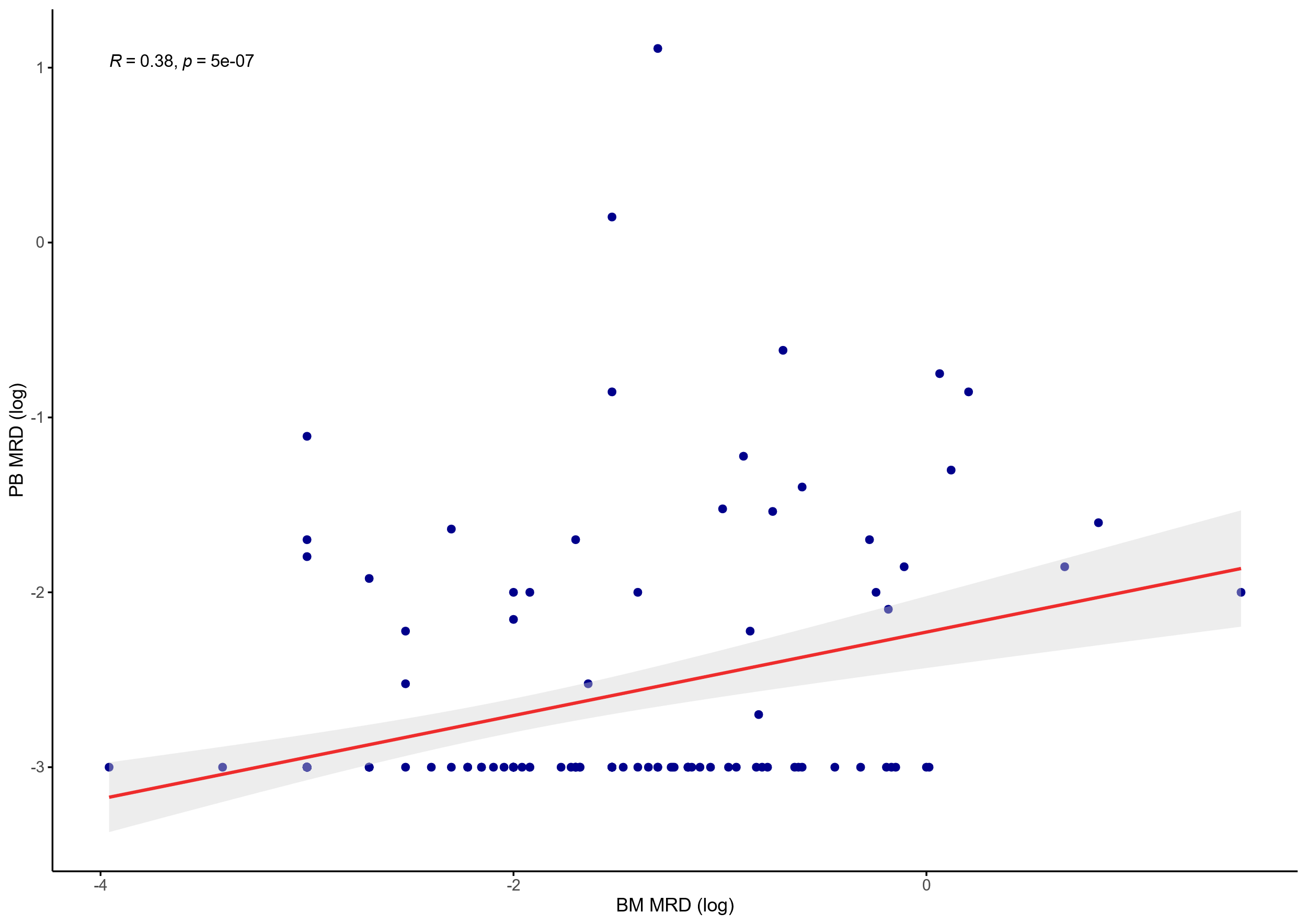
**

**C.**


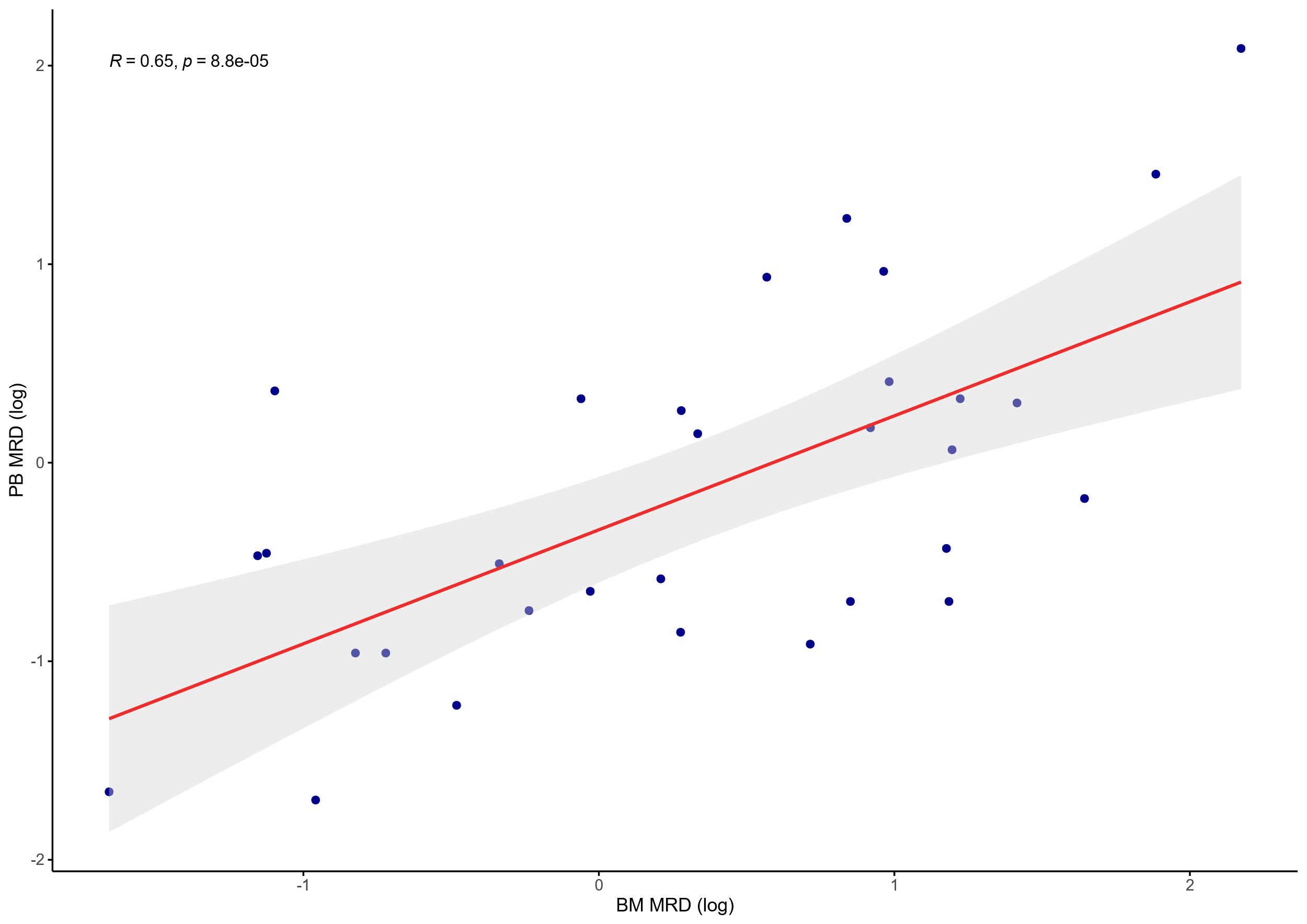


**Supplementary Figure 3.** (A) Cumulative incidence of molecular and morphologic relapses in patient with CR_MRD-_ (n=105), CR_MRD-LL_ at low level (n=66), or CR_MRD+_ other than CR_MRD-LL_ (n=92) in bone marrow after first-line therapy; (B) Cumulative incidence of molecular and morphologic relapses in patient with CR_MRD-_ (n=148), CR_MRD-LL_ at low level (n=18), or CR_MRD+_ other than CR_MRD-LL_ (n=22) in peripheral blood after first-line therapy.

**A.**

**
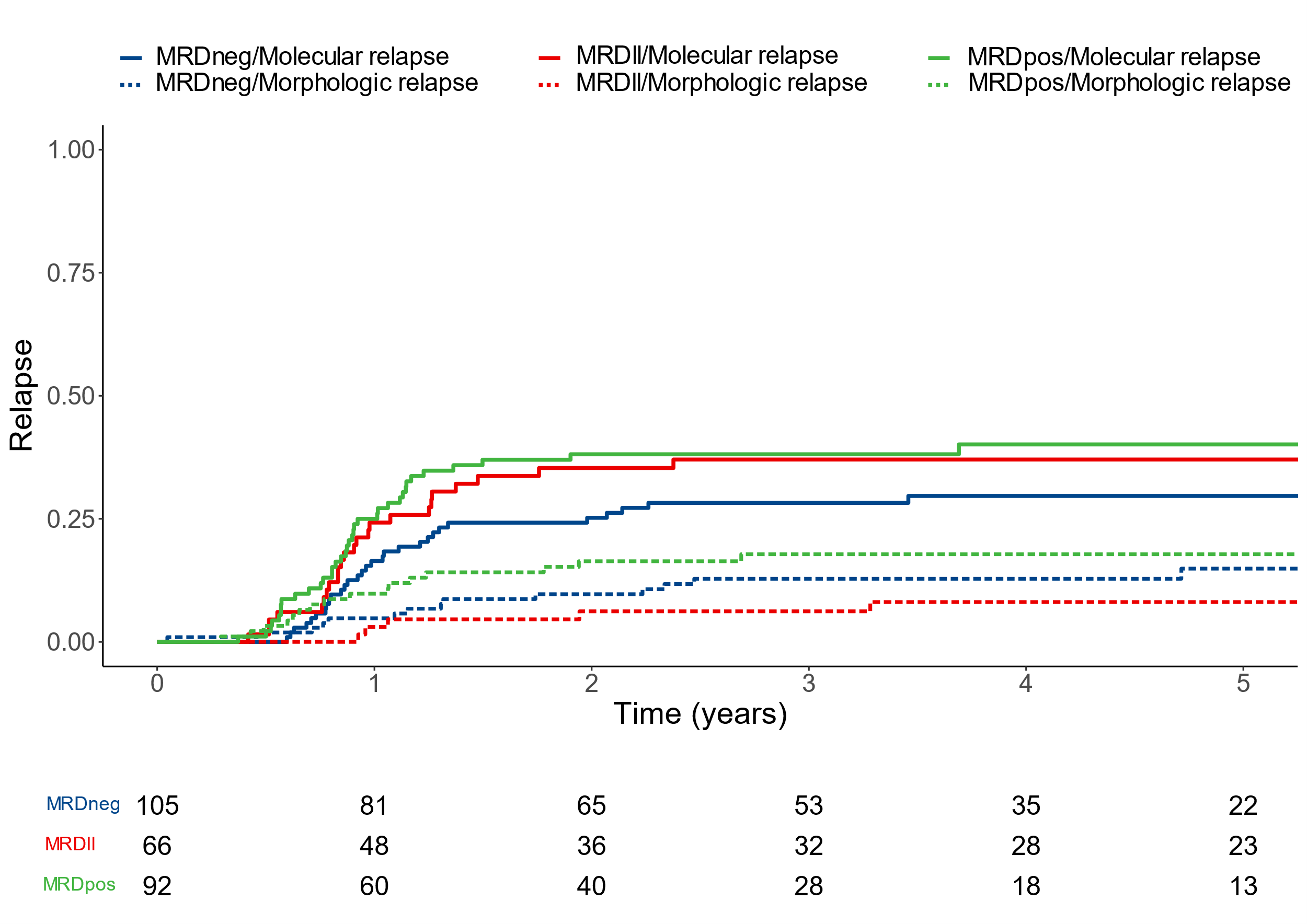
**

**B.**

**
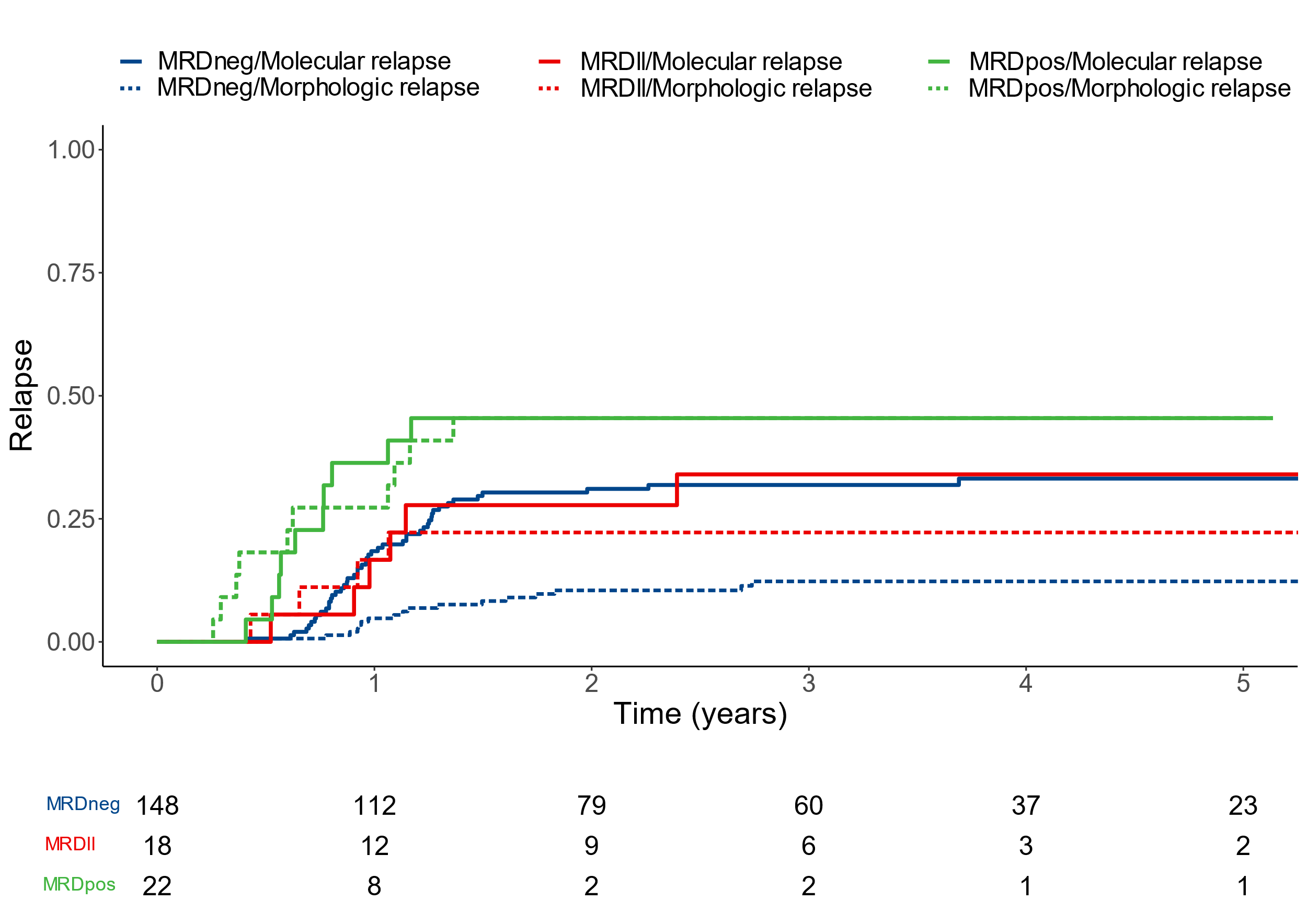
**

**Supplementary Figure 4.** (A) Overall survival of 61 patients with CBF AML stratified by the type of relapse (molecular relapse with preemptive therapy *vs.* molecular relapse with morphologic relapse at the time of salvage therapy *vs.* upfront morphologic relapse), calculated from the time of first detected relapse; (B) Overall survival of 88 patients with *NPM1*-mutated AML, stratified by the type of relapse (molecular relapse with preemptive therapy *vs.* molecular relapse with morphologic relapse at the time of salvage therapy *vs.* upfront morphologic relapse), calculated from the time of first detected relapse.

**A.**


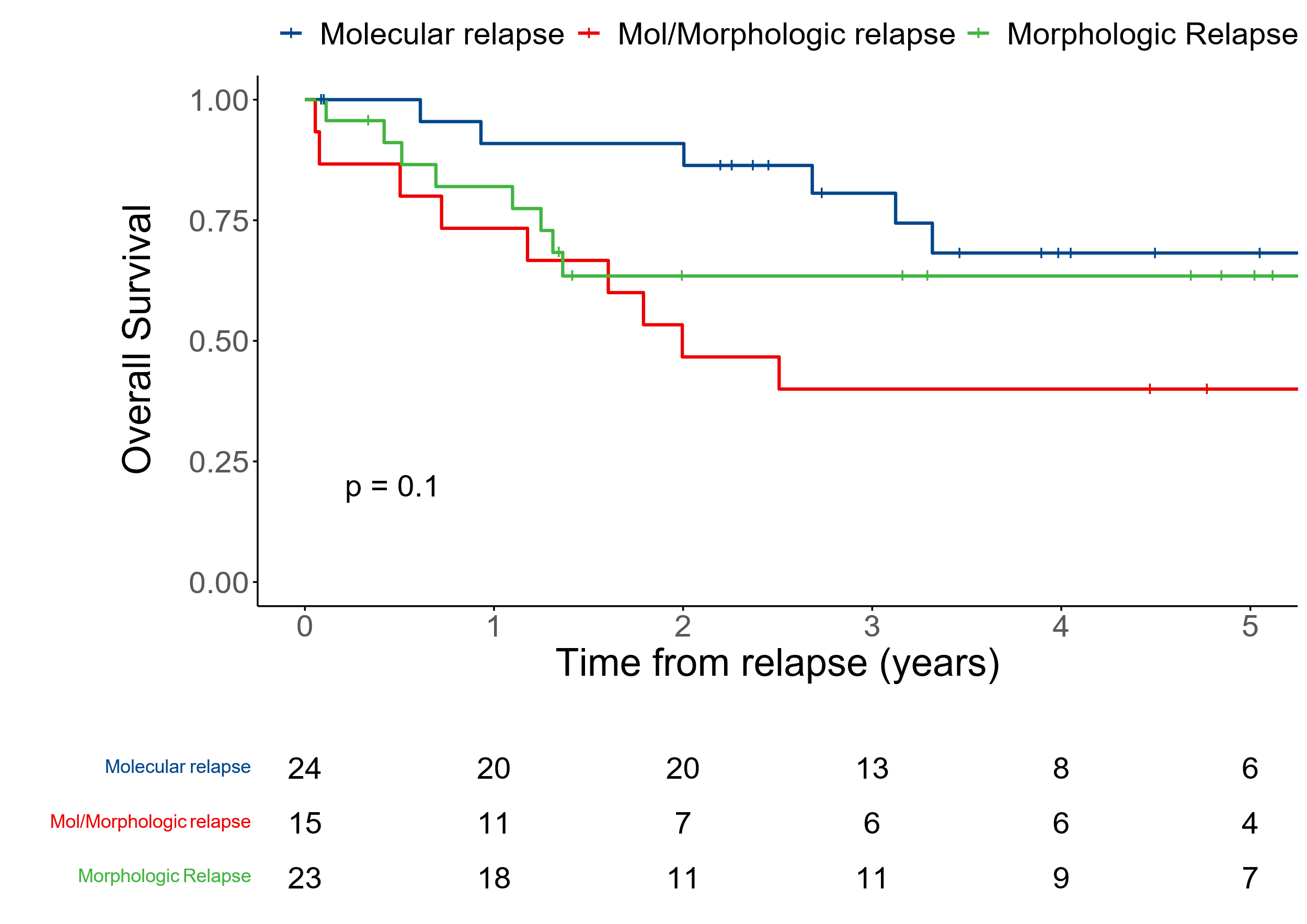


**B.**


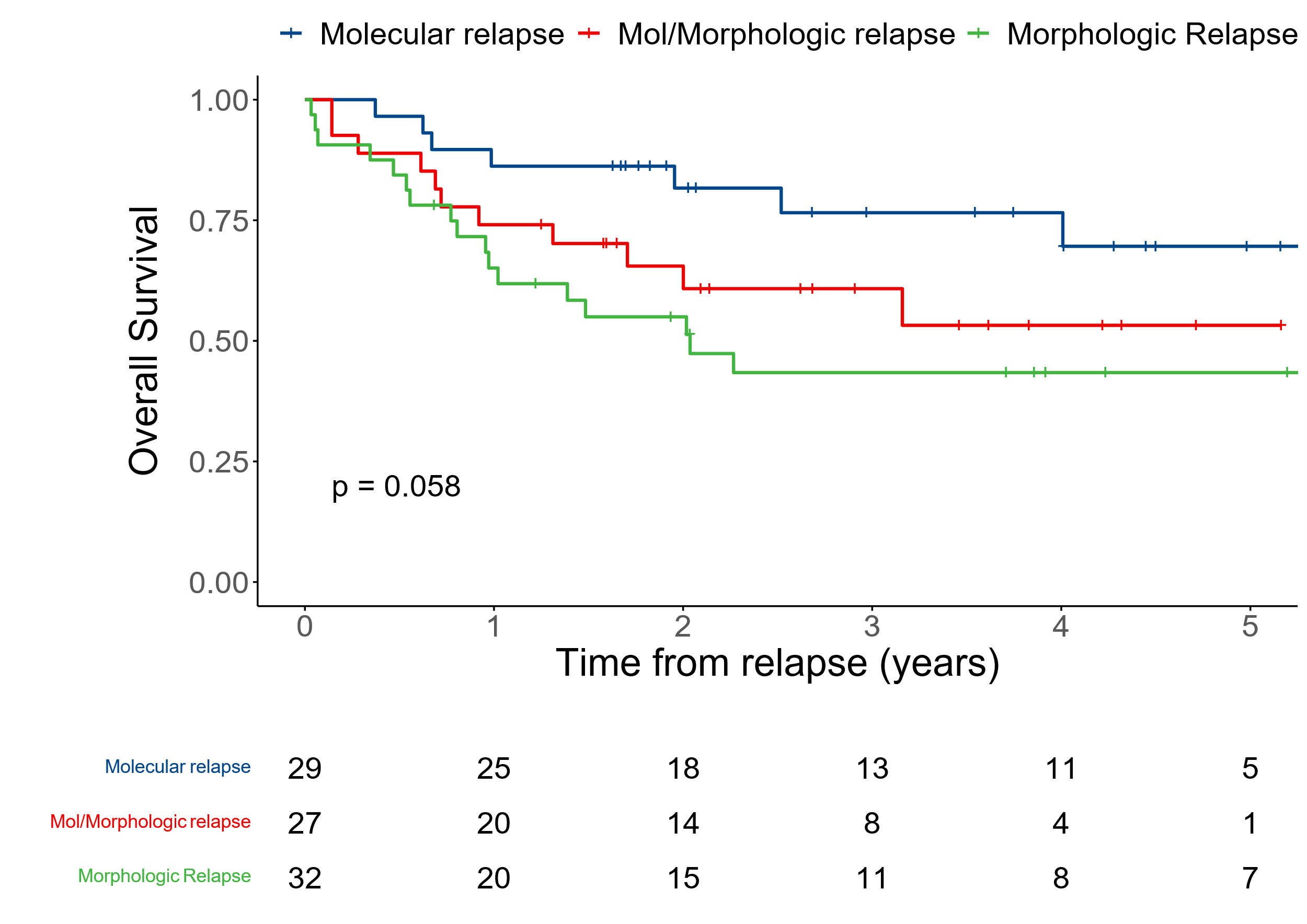


**Supplementary Figure 5.** Overall survival of 148 patients with CBF or *NPM1*-mutated AML who relapsed during follow-up and received salvage therapy, stratified by the type of relapse (molecular relapse with preemptive therapy *vs.* molecular relapse with morphologic relapse at the time of salvage therapy *vs.* upfront morphologic relapse), calculated from the time of salvage therapy initiation.


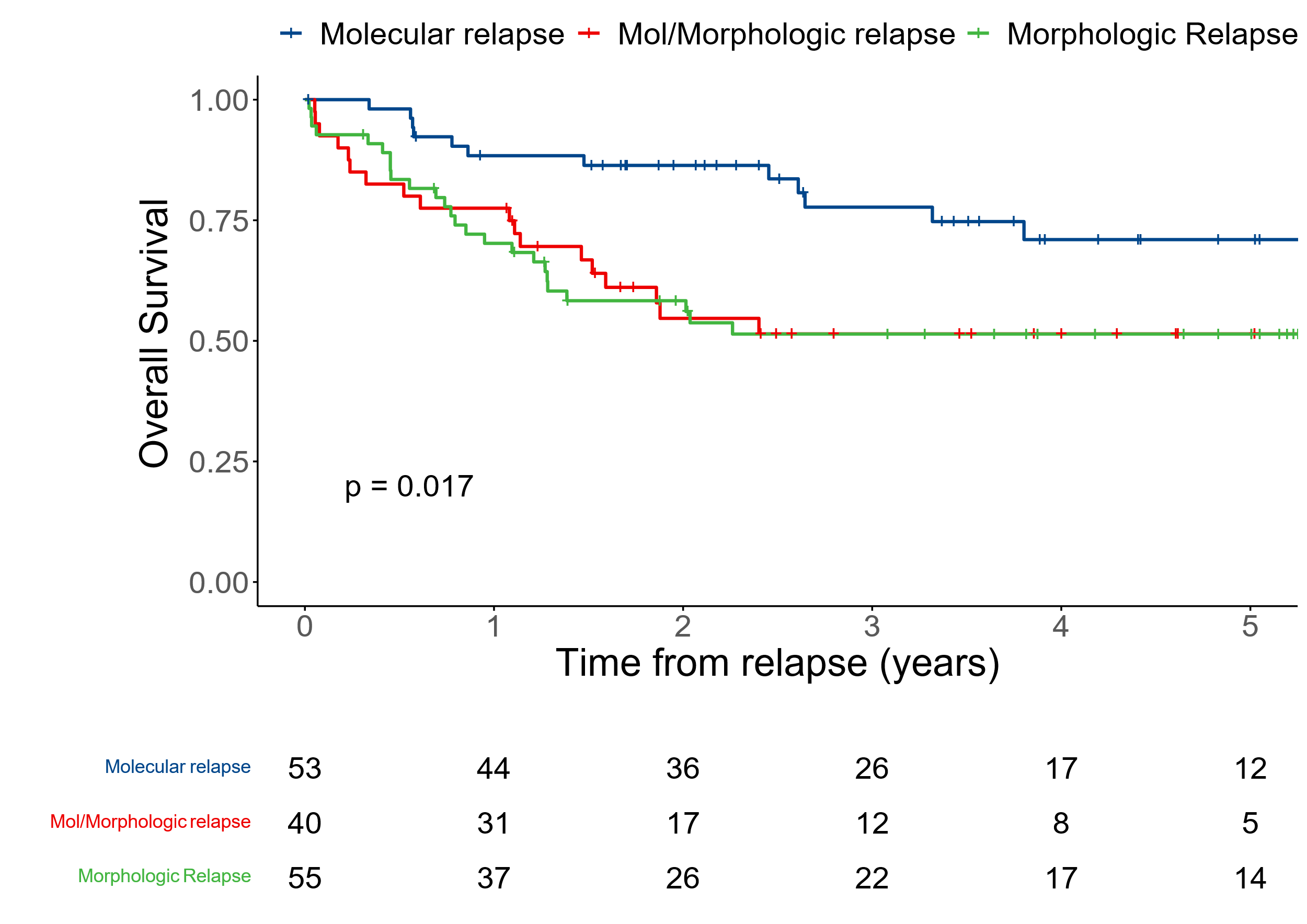


**Supplementary Figure 6.** Treatment-related mortality of 150 patients with CBF or *NPM1*-mutated AML who relapsed during follow-up, stratified by the type of relapse (molecular relapse with preemptive therapy *vs.* molecular relapse with morphologic relapse at the time of salvage therapy *vs.* upfront morphologic relapse), calculated from the time of first detected relapse.


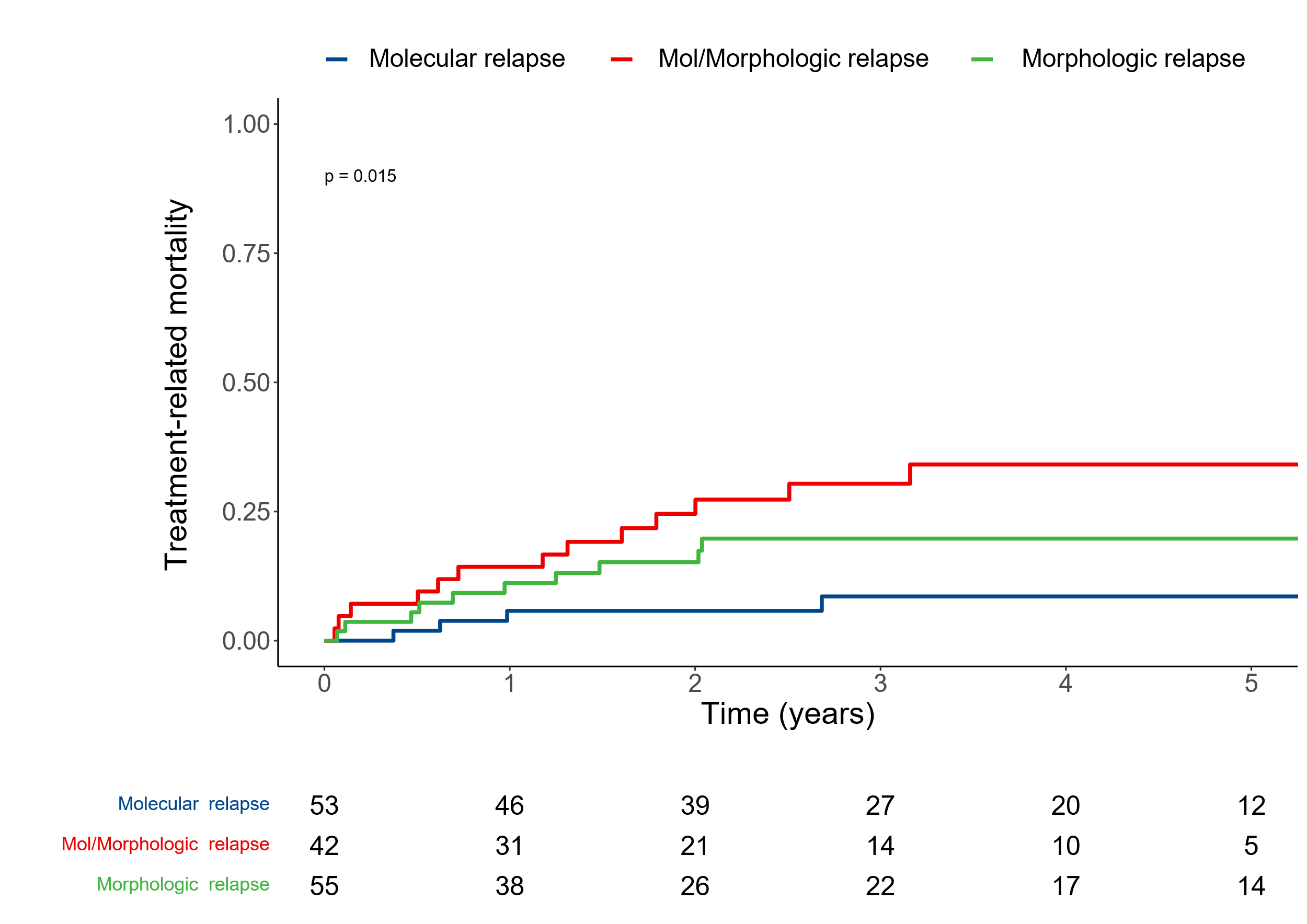


**Supplementary Figure 7.** Overall survival of 150 patients with CBF or *NPM1*-mutated AML who relapsed during follow-up, stratified by allogeneic HCT as part of salvage therapy, calculated from the time of first detected relapse.


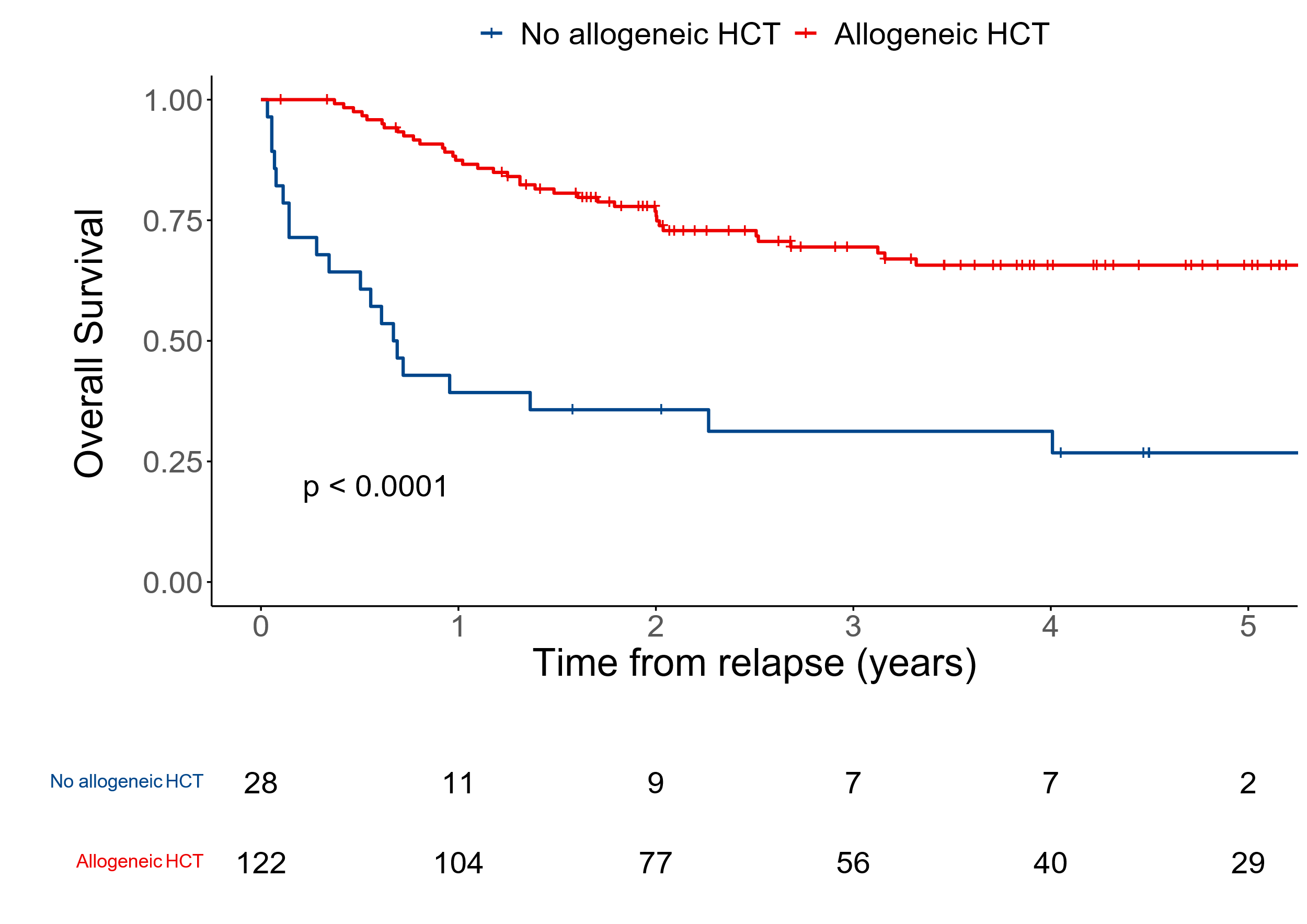

Supplement: Supplementary file 1 — Supplemental material [file 41375_2024_2335_MOESM1_ESM.docx]
